# Identification of Bioactive Medium Chain Fatty Acids (C_10_, C_8_, and C_6_) in Ozonated Sunflower Oil: Comparative Evaluation of Their Potent Antioxidant Activities and Anti-Inflammatory Effects in the Hyperinflammatory Zebrafish Model

**DOI:** 10.3390/antiox15050606

**Published:** 2026-05-10

**Authors:** Kyung-Hyun Cho, Kyoung Ah Min, Krismala Djayanti, Yunki Lee, Sang Hyuk Lee, Yassmine Benmokadem, Ashutosh Bahuguna

**Affiliations:** 1 Raydel HDL Research Institute, Medical Innovation Complex, Daegu 41061, Republic of Korea; 2 College of Pharmacy and Inje Institute of Pharmaceutical Sciences and Research, Inje University, Gimhae 50834, Republic of Korea

**Keywords:** antioxidant, dyslipidemia, decanoic acid, hexanoic acid, octanoic acid, ozonated sunflower oil, paraoxonase, senescence, steatosis

## Abstract

Three medium-chain fatty acids (MCFAs), namely decanoic acid, octanoic acid, and hexanoic acid, were identified in ozonated sunflower oil (OSO) using high-performance liquid chromatography (HPLC) and liquid chromatography-mass spectrometry (LC/MS). All three MCFAs exhibited strong in vitro antioxidant activity to enhance high-density lipoprotein (HDL)-associated paraoxonase and protected low-density lipoproteins (LDL) from oxidative damage caused by Cu^2+^ ions. Consistently, MCFAs displayed substantial cellular antioxidant activity and minimized carboxymethyllysine (CML)-induced reactive oxygen species (ROS) generation and apoptotic cell death in zebrafish embryos. In adult zebrafish, MCFAs treatment mitigated CML-induced acute death and swimming abnormalities, and substantially augmented plasma sulfhydryl content, ferric ion reduction ability (FRA), and paraoxonase (PON)-like activity. Also, MCFA-treated zebrafish showed lower blood glucose, total cholesterol (TC) and triglycerides (TG) with raising HDL cholesterol levels. The MCFAs showed substantial inhibition of hepatic ROS generation, neutrophil efflux, interleukin (IL)-6 production, and steatosis, leading to hepatoprotection against CML-triggered adversity. Consistent with hepatic histology results, reduced plasma hepatic function biomarkers aspartate aminotransferase (AST) and alanine aminotransferase (ALT) levels were observed in MCFA-treated groups than in the CML-treated group. In the kidney, MCFA treatment effectively reduced oxidative stress and cellular senescence and protected against kidney damage induced by exposure to CML. The study concludes the presence of three MCFAs in the OSO, which serve as functional antioxidants and anti-inflammatory agents, accounting for its diverse pharmacological properties.

## 1. Introduction

Ozone is an important gas with several beneficial health effects [1,2,3]. However, its short lifetime is always a matter of concern, which can be substantially elevated by the insertion of ozone into water and a variety of vegetable oils. Ozone insertion into water and oil produces various ozone-derived compounds, including ozonides, peroxides, and carboxylic acids [4]. Unlike ozone gas, ozonated oils do not contain free ozone but instead consist of these secondary products, which are responsible for their prolonged biological activities [4].

Since the first article about ozonated oil with turpentine appeared in 1854, there have been more than 850 published articles in PubMed with the searched terms of ozonated oil until 2 March 2026 [5,6]. In the global market, many products containing ozonated oils have appeared using a variety of oils, including hemp seed oil, sunflower oil (SO), olive oil, coconut oil, etc., with various formulations such as liquid, cream, and salve [7,8]. Many topical and oral applications of ozonated oils have been developed for wide medical and cosmetic use in the field of dermatology, dentistry, ophthalmology, and gynecology [7]. Among the many ozonated oils, ozonated sunflower oil (OSO) has demonstrated substantial efficacy as an alternative or complementary therapeutic agent for various infectious diseases, inflammatory dermal diseases, antibacterial, antifungal, and antiparasitic activities, and dermatological disorders [4,7,9]. The potent antimicrobial and antioxidant effects of OSO protected cells and zebrafish embryos against oxidative stress, with a notable impact on the activity enhancement of high-density lipoprotein (HDL)-associated antioxidant paraoxonase (PON) [10]. In addition, bactericidal, fungicidal, and wound-healing activities in dermal applications [11], as well as intraperitoneal and oral consumption of OSO, have been shown to improve blood antioxidant activity, lipid profile, and tissue regeneration in zebrafish and rat models [12,13,14]. In addition, many promising results of OSO have been reported for the treatment of burns [15], atopic dermatitis (AD) [16], tinea pedis [17], and pressure ulcers [18].

Despite its wide pharmacological and cosmetic applications, the active ingredients in OSO that mediate tissue regeneration, anti-inflammatory, and germicidal activities are still not fully understood. However, formation of ozonides, hydroperoxides, and aldehydes has been recognized as key constituents of OSO broadly responsible for many functionalities; still, there is scarce information about hidden and unidentified substances formed by the interaction between ozone and unsaturated fatty acids [19]. Notably, OSO has a distinct pungent smell, such as a citrus-like rancid odor, which does not occur in SO, indicating the presence of certain volatile organic acids, which might be short-chain carboxylic acids, in OSO formed during the ozonide reaction.

Based on the aforementioned assumption, in an attempt to identify active ingredients, three medium-chain fatty acids (MCFAs), viz., decanoic acid, octanoic acid, and hexanoic acid, were targeted in OSO and compared with SO using high-performance liquid chromatography (HPLC) and liquid chromatography-mass spectrometry (LC/MS). Furthermore, the comparative antioxidant potential of these MCFAs was evaluated in vitro. Additionally, the comparative efficacy of these MCFAs against carboxymethyllysine (CML)-induced oxidative stress, inflammation, and hepatorenal damage was investigated in a zebrafish model.

Zebrafish were chosen as a model organism due to their notable similarities to humans in several organ development and physiological functions [20]. Interestingly, zebrafish share key features of lipid metabolism with humans, including comparable receptors, lipoproteins, and metabolic enzymes [21]. Importantly, zebrafish possess cholesteryl ester transfer protein (CETP) [22], which facilitates cholesterol transport between HDL and LDL/VLDL, a feature absent in mice and rats [23]. Furthermore, many cell types, tissues and disease-related pathways in zebrafish closely resemble those in humans [24]. Unlike most rodent models, zebrafish’s small size (~3–5 cm) [25], cost-effectiveness, and ease of maintenance allow studies with large sample sizes in limited space. Due to these advantages, zebrafish have gained considerable attention as an efficient model for studying various human diseases [24,26,27], and outcomes from the zebrafish studies can provide valuable insights in shaping human studies.

## 2. Materials and Methods

### 2.1. Materials

Ozonated sunflower oil (OSO) and sunflower oil (SO) were complementary and provided by the National Center for Scientific Research (CNIC), Havana, Cuba. The refined SO used has the iodine value 130 g I_2_/100 g, saponification value 191.1 mg KOH/g, peroxide value ≤ 15 mmol-equiv/kg and contains fatty acids such as myristic acid (≤0.1%), palmitic acid (5.1%), stearic acid (4.3%), oleic acid (52.3%), linoleic acid (38.1%). The SO was free of pathogenic microbial contamination, including *Staphylococcus aureus*, *Escherichia coli*, *Pseudomonas aeruginosa*, *Candida albicans*, and *Salmonella* sp. For the preparation of OSO, ozone gas (~100 g/m^3^) was pumped into the reactor containing SO, which was maintained at 20 ± 5 °C. The ozonation process is continued (~2 h) to attain the peroxide value 700–850 mmol-equiv/kg. After achieving the peroxide value (700–850 mmol-equiv/kg), the ozonation process was stopped, and the formed OSO was left at room temperature. After 12 h, the peroxide value was recalculated, and if the peroxide value falls in the range 500–800 mmol-equiv/kg, the sample is passed for further quality control and stored at 5 ± 3 °C. The used OSO had a peroxide value of 746.2 mmol-equiv/kg, acidity of 2.45 mg KOH/g, aldehyde concentration of 0.24 mmol/g, viscosity of 110.35 mPa.s, and was free of pathogenic microbes. A detailed specification of the OSO is provided as a Supplementary Table S1. Decanoic acid (C1875-100 g), octanoic acid (21639-5 mL), hexanoic acid (21529-5 mL), heptadecanoic acid (H-3500-5 g), methyl *tert*-butyl ether, *N*-(3-dimethylaminopropyl-*N*′-ethylcarbodiimide hydrochloride (E1769-5 g), pyridine (360570-100 mL) and carboxymethyllysine (cat:14580-5 g) were purchased from Sigma Aldrich (St. Louis, MO, USA). 2-Nitrophenylhydrazine hydrochloride (NPH; N0231) was purchased from Tokyo Chemical Industry (Tokyo, Japan). Acetonitrile and methanol (LC/MS grade) were purchased from Honeywell Burdick & Jackson (Morristown, NJ, USA). All the other chemicals were of analytical grade and used as supplied unless otherwise stated.

### 2.2. Extraction of Oil Samples for HPLC and LC/MS Analysis

Sunflower oil (SO) and ozonated sunflower oil (OSO) samples were extracted with methanol/methyl *tert*-butyl ether (1:3, *v*/*v*) for HPLC and LC/MS analysis, as previously reported [28]. The oil sample, 2 g of SO or OSO, was mixed with 5 mL of methanol/methyl *tert*-butyl ether (1:3, *v*/*v*), then homogenized for 3 min. Next, 5 mL of methanol/methyl *tert*-butyl ether (1:3, *v*/*v*) was added, and the mixture was vortexed for 5 min followed by 10 min of sonication in a cold-water bath (4 °C). Then, 5 mL of water/methanol (3:1, *v*/*v*) was added, and the mixture was vortexed for 10 min. The mixture was centrifuged at 12,000 rpm for 10 min at 4 °C to achieve phase separation. The lower phase was carefully collected and transferred to a new tube. After the heptadecanoic acid (an internal standard) was added, the mixture was vortexed thoroughly. The solvent was evaporated using a rotatory vacuum evaporator at 50 °C. The dried residue was reconstituted in 2 mL of acetonitrile/methanol (1:1, *v*/*v*). Finally, the solution was filtered through a 0.22 μm syringe-type nylon membrane filter. The samples were stored at −70 °C until further analysis by the HPLC. For LC/MS analysis, the extracted oil sample solution was reconstituted in 2 mL of acetonitrile/methanol (1:1, *v*/*v*) containing 0.1% formic acid and diluted further with the reconstitution solvent for LC/MS analysis to achieve concentrations within the calibration standard range for each fatty acid.

### 2.3. Preparation of Calibration Standard Solution for HPLC and LC/MS Analysis

A stock solution was prepared by dissolving a weighed amount (10 mg) of each reference standard of MCFAs (hexanoic acid, octanoic acid, or decanoic acid) in 1 mL of methanol. The resulting 10 mg/mL standard solution was filtered through a 0.22 μm nylon syringe filter (Derian, Shanghai, China, Cat. No. SN01322PTFE-D). Calibration standard solutions were prepared by diluting 1 mg/mL of standard stock solutions with the diluent solvent (acetonitrile:methanol, 1:1, *v*/*v*) to the desired concentration range. The final concentration ranges of the calibration standards of each compound (hexanoic acid, octanoic acid, or decanoic acid) for HPLC analysis were 0.5 to 7.5 mg/mL. For LC/MS analysis, the standard stock and diluted calibration solution were prepared in acetonitrile/methanol (1:1, *v*/*v*) with 0.1% formic acid. The final concentration ranges of the calibration standards of each compound (hexanoic acid, octanoic acid, or decanoic acid) for LC/MS analysis were 0.5 to 1000 μg/mL. Three independent batches of calibration standards were prepared and used for preparing calibration curves.

### 2.4. HPLC and LC/MS Analysis of Medium Chain Fatty Acid Standards and Oil Samples

The standard solution and extracted oil sample solution were analyzed using the Alliance^®^ 2695 HPLC system equipped with a quaternary pump, a thermostatic autosampler, and a 2487 dual UV absorbance detector (Waters, Milford, MA, USA). The HPLC system was controlled by the Empower 2 software. For sample analysis, 20 μL of solution was injected into the HPLC system, and separation was carried out on the Kinetex™ C8 column (4.6 mm i.d. × 250 mm, 5 μm particle size, 100 Å pore size; Phenomenex, Torrance, CA, USA) in the column oven with a set temperature of 40 °C. The mobile solution A was prepared by mixing acetonitrile and methanol in a 1:1 (*v*/*v*) ratio, while the mobile solution B was prepared by mixing acetonitrile and methanol at a 3:1 (*v*/*v*) ratio. Each mobile solvent was degassed by ultrasonication followed by vacuum treatment prior to use. The chromatographic method of the analysis was performed using the mobile solvent gradient at the flow rate of 0.6 mL/min as follows: 85% mobile B at 0 min; held at 85% mobile B for 1 min; linear gradient from 1 to 2 min up to 95% B; linear gradient from 2 to 3 min up to 100% B; held at 100% B from 3 to 25 min; returned to initial 85% B from 25 to 28 min; and held at 85% B from 28 to 30 min. The signals of the MCFAs (hexanoic acid, octanoic acid, or decanoic acid) were measured at 214 nm wavelength. For the quantification of MCFAs, a regression equation derived from a variety of known concentrations of standard hexanoic acid, octanoic acid, or decanoic acid was utilized, as mentioned in Supplementary Table S2.

Furthermore, the standard solution and the extracted oil sample solution were analyzed using Agilent^®^ 1200 Infinity liquid chromatography coupled to an Agilent^®^ 6475 triple quadrupole mass spectrometer with an Agilent Jet Stream source in positive or negative ESI mode (Agilent Technologies, Santa Clara, CA, USA). The LC/MS system was controlled using Agilent Masshunter 12.1 ver. software. For the analysis, 5 μL of sample solution was injected into the LC/MS, and separation was carried out on the InfinityLab Poroshell™ 120 EC-C8 column (3.0 mm i.d. × 50 mm, 2.7 μm particle size; Agilent Inc., Santa Clara, CA, USA) in the column oven at 40 °C. The mobile solution A consisted of water with 0.1% formic acid, while the mobile solution B consisted of acetonitrile/methanol (1:1, *v*/*v*) with 0.1% formic acid. Each mobile solvent was degassed prior to use. The isocratic chromatographic method using mobile solvent A/B (5:95, *v*/*v*) was applied at a flow rate of 0.4 mL/min for 12 min. The mass spectrometer of LC/MS was operated with the ESI source set as follows: gas temperature at 300 °C, sheath gas temperature at 350 °C, gas flow at 13 L/min, sheath gas flow at 11 L/min, nebulizer gas pressure at 35 psi, capillary voltage at 4 kV, and nozzle voltage at 1.5 kV.

### 2.5. NPH Derivatization of Standard and Oil Samples and HPLC Analysis of Derivatized Samples

The 2-nitrophenylhydrazine hydrochloride (NPH) derivatization and extraction procedures were performed according to the previously reported methods [29,30]. In brief, standard solutions (100 μL) of each reference standard of MCFAs (hexanoic acid, octanoic acid, decanoic acid) at the varied concentrations (5–125 μg/mL) were mixed with 100 μL of methanol and vortexed for 30 s. Also, 35 μL of SO or OSO was mixed with 200 μL of methanol and vortexed for 30 s. In the MCFAs, SO and OSO solution, 200 μL of a freshly prepared 0.02 M of NPH dissolved in 40 mM HCl/ethanol (3:1, *v*/*v*) and 400 μL of 0.25 M of *N*-(3-dimethylaminopropyl-*N*′-ethylcarbodiimide hydrochloride solution (prepared in 3% pyridine in ethanol) was added. After 30 s of vortexing, the reaction mixture was incubated at 60 °C for 20 min for derivatization. Thereafter, 100 μL of 15% KOH solution (prepared in a 80% methanol) was added. The mixture solution was incubated at 60 °C for 15 min following the addition of 4 mL solution [of 0.03 M phosphate buffer (pH 6.4)/0.5 M HCl (3.8:0.4, *v*/*v*)]. Finally, for the extraction process, 4 mL of methyl *tert*-butyl ether containing the internal standard (heptadecanoic acid) was added to the mixture, which was then vortexed vigorously for 5 min. The solution was centrifuged at 12,000 rpm for 10 min. The organic (upper) layer was carefully collected and evaporated to dryness using an evaporator preheated to 50 °C. The dried residue was reconstituted in 200 μL of acetonitrile/methanol (1:1, *v*/*v*) and vortexed for 3 min to ensure complete dissolution. The reconstituted solution was filtered through a 0.22 μm nylon syringe filter and processed for HPLC under gradient elution conditions as explained in Supplementary Method Section S1.

### 2.6. Ferric Ion Reduction Ability (FRA) of MCFAs

For the detection of FRA, 20 μL of MCFAs (at final concentration 5, 20, and 50 μM) were mixed with 180 μL of FRA reagent [prepared by mixing 0.3 M sodium acetate with 0.01 M of 2,4,6-tripridyl-*S* triazin and 0.02 M ferric chloride (10:1:1 *v*/*v*)]. After 30 min of incubation at room temperature, absorbance at 593 nm was measured using a spectrophotometer.

### 2.7. Effect of MCFAs on HDL Associated Paraoxonase Activity and LDL Oxidation

#### 2.7.1. Isolation of LDL and HDL

Blood was voluntarily donated by a healthy male individual (25 ± 3 years old) after 12 h of fasting, following the Helsinki guidelines approved by the Institutional Board of the Korea National Institute for Bioethics Policy (KoNIBP, approval number P01-202109-31-009, approval date 27 September 2021). LDL and HDL from blood were isolated by sequential ultracentrifugation using the method described earlier [13]. In brief, 5 mL of serum was mixed with density grade 1.019 < d < 1.063 (for LDL) and 1.063 < d < 1.225 (for HDL). The density gradient was prepared with NaCl and NaBr according to the standard protocol [31]. The content was centrifuged at 100,000× *g* for 24 h, and the separated LDL and HDL were isolated from their respective density zones. Individually, LDL and HDL were processed for dialysis using Tris-buffered saline (TBS, pH 8.0) in a cool atmosphere (4 °C) to remove the salts. After 24 h, the dialyzed LDL and HDL were recovered and stored in a refrigerator for further use.

#### 2.7.2. Paraoxonase (PON) Activity in High-Density Lipoprotein (HDL)

The PON activity in HDL was quantified using the earlier described method [13]. In brief, 20 μL MCFAs (50 μM) were mixed with 20 μL of HDL (1 mg/mL), followed by the addition of 160 μL of 0.55 M paraoxon-ethyl substrate. After 60 min incubations at 25 °C, absorbance at 415 nm was recorded, and the activity was expressed as μU/L/min using the molar absorbance coefficient (1.7 × 10^4^ M^−1^ cm^−1^) of the formed product *p*-nitrophenol.

#### 2.7.3. Low-Density Lipoprotein (LDL) Oxidation and Quantification of LDL-Oxidized Products

The oxidation of LDL was caused by the exposure to Cu^2+^ using the method described earlier [32]. In brief, 75 μL of LDL (1.0 mg/mL) was mixed with 25 μL of CuSO_4_ (10 μM) containing 50 μM MCFAs (either decanoic, hexanoic, or octanoic acid). Likewise, LDL + CuSO_4_ without MCFAs was used as the control. The content was incubated at 37 °C for 2 h, followed by agarose (0.5%) gel electrophoresis using the Tris-acetate-EDTA buffer (pH 8.0). After ~1 h (at 50 V), electrophoresis was stopped, and the gel was stained with 1.25% Coomassie Brilliant Blue (CBB).

The extent of oxidation in LDL following exposure to Cu^2+^ and MCFAs (as mentioned earlier) was quantified using the thiobarbituric acid reactive substances (TBARS) assay [32]. In brief, 50 μL of LDL-containing samples were mixed with 50 μL of 20% trichloroacetic acid (TCA) and 100 μL of 0.57% thiobarbituric acid. After 10 min of incubation at 95 °C, the absorbance at 560 nm was measured, and results were expressed as the amount of malondialdehyde (MDA) formed.

### 2.8. Zebrafish Rearing

Adult zebrafish were maintained in the circulating water (28 °C) under a 14 h light and 10 h dark photoperiod following the standard guidelines for Animal Care and Use as adopted by Raydel Research Institute (approval no. RRI-24-001, date of approval 2 September 2024). The water used to culture zebrafish was free of the coliform group of microorganisms, with a total bacterial load of ≤100 CFU, pH 7.3, turbidity 0.16 NTU, residual chlorine 0.18 mg/L, and dissolved oxygen 8 mg/L. The water was certified as safe for use by animals and humans based on the water analysis conducted by Kirim Life Science Co., Ltd., Daegu, Republic of Korea (Supplementary Table S3).

Zebrafish were fed with a normal tetrabit commercial fish diet (Tetrabit GmbH, D49307, Melle, Germany) twice a day (9 am and 6 pm). Zebrafish were acclimatized under the same conditions for one week prior to any additional experiments.

### 2.9. Zebrafish Embryo Production and Microinjection

Female (two) and male (one) zebrafish were placed in the breeding tank and separated from each other using a divider. In the morning (~8.30 am), the divider was removed, and male and female zebrafish were allowed to mate uninterrupted in the dark. After 30 min of mating, embryos were collected, washed with water, and preserved in 0.01% (*w*/*v*) sea saltwater solution containing 0.1 mg/mL methylene blue.

Embryos (1.5 h post-fertilization) were segregated into five groups (*n* = 150/group). Zebrafish received a 10 nL PBS microinjection in the PBS group. Whereas 10 nL microinjection of CML (final 500 ng in PBS) was injected into the CML-injected group. Similarly, 10 nL of PBS containing CML (500 ng) was microinjected in the presence of either 2 ng decanoic acid, octanoic acid, or hexanoic acid, and the groups were named decanoic acid-, octanoic acid-, and hexanoic acid-treated groups. Embryos across the groups were monitored for up to 72 h post-treatment to assess survival and developmental deformities. The selected dose (2 ng) was determined from preliminary screening experiments in which individual MCFAs were tested at concentrations ranging from 0.25 to 10 ng (*n* = 20 embryos/group) in the presence of CML (500 ng). Post-24 h treatment, embryo survivability was assessed, revealing a concentration-dependent increase up to 1.5 ng for hexanoic and octanoic acid, with no significant change beyond this range. In contrast, decanoic acid showed increased survival up to 2 ng, which remained statistically similar at higher amounts up to 10 ng. To ensure consistency and enable comparative analysis, an amount of 2 ng was selected as the minimum dose yielding maximal protective effect. A CML (500 ng) was selected based on the previously optimized dose [33].

### 2.10. Dihydroethidium (DHE) and Acridine Orange (AO) Staining

Ten embryos from each group were placed in separate wells of a 24-well plate. Subsequently, a 500 μL solution of DHE (30 μM) and AO (5 μg/mL) was applied. After 30 min incubation in the dark at room temperature, solutions from each well were decanted, and embryos were washed (3 times) with PBS and visualized under the fluorescent microscope (Nikon Eclipse TE2000, Tokyo, Japan). DHE and AO fluorescence were detected as 585 nm and 505 nm excitation wavelengths and 615 nm and 535 nm emission wavelengths, respectively.

### 2.11. Acute Toxicity in Adult Zebrafish

**Figure 1:**
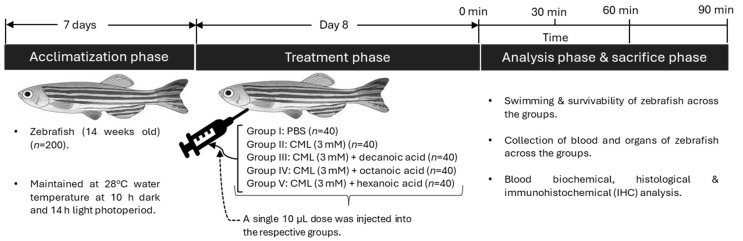
Schematic representation of the experimental plan. PBS: phosphate-buffered saline; CML: carboxymethyllysine.

Adult zebrafish (*n* = 200) were randomly divided into five groups (*n* = 40/group). In the PBS group, zebrafish received a 10 μL intraperitoneal injection of PBS. Likewise, the zebrafish in the CML groups were intraperitoneally injected with 3 mM CML dissolved in 10 μL of PBS. Zebrafish in decanoic acid, octanoic acid, and hexanoic acid-treated groups received a 10 μL intraperitoneal injection of PBS containing CML (3 mM) with 5 μg of decanoic acid, octanoic acid, and hexanoic acid, respectively (Figure 1). The dose (5 μg) was selected based on preliminary experiments in which MCFAs were administered intraperitoneally at doses ranging from 2.5 to 10 μg in the presence of CML, and survivability was assessed 90 min post-treatment. All MCFAs showed a dose-dependent increase in survivability, with a maximum at 5 μg, whereas doses above 5 μg did not provide any additional significant protective effect. Therefore, 5 μg was chosen as the minimum dose that achieved optimal protection for all subsequent experiments. CML (3 mM) was selected based on the previously optimized dose [33].

For each group (*n* = 40), zebrafish were divided into 4 tanks (10 fish/tank × 4 tanks). Zebrafish in the distinct tanks were observed for survival and swimming activity at 5 min, 30 min, 60 min, and 90 min of treatment.

Swimming activity was examined by visualizing tail fin movement and the paucity of body paroxysm [34]. Zebrafish mortality was assessed by observing head-up or head-down posture, floating at the surface or sinking to the bottom of the tank, and examining gill movements, following OECD 2019 guidelines [35].

### 2.12. Collection of Blood and Organs

After 90 min of treatment, zebrafish (*n* = 40) across the groups were sacrificed using hypothermic shock to collect blood (2–5 μL/fish). Blood was collected separately from the zebrafish maintained in the four different tanks for each group (as described in Section 2.11). Blood from the zebrafish (*n* = 10/tank) of the specific group was collected and pooled in a single tube and subsequently mixed with ethylenediaminetetraacetic acid (EDTA, 1 mM) to 2 ratio 3 (2:3) and processed by centrifugation at 6000 rpm for 10 min. The supernatant was collected and kept in a refrigerator (4 °C) for biochemical analysis. Notably, if any fish died before completing the 90 min of treatment, blood was collected immediately after death was confirmed. The collected blood was then pooled with the blood obtained at the end of 90 min treatment from zebrafish belonging to the same experimental group and maintained in a similar tank.

Liver and kidney from the zebrafish were retrieved surgically under the stereomicroscope (Motic SMZ 168, Hong Kong, China). The organs were preserved in 10% formalin for further histological analysis.

### 2.13. Plasma Levels of Lipoproteins, Glucose, Hepatic Function Biomarkers, and Oxidative and Antioxidant Variables

Plasma TC (AM 202-K), TG (AM 157-K), HDL-C (AM 203-K), AST (AM 102K), and ALT (AM 103-K) levels were quantified using commercial assay kits from Asan Pharmaceutical, Hwasung, Republic of Korea. A detailed methodology is provided in Supplementary Method Section S2. A digital blood glucose meter (AccuCheck, Roche, Basel, Switzerland) was utilized to quantify the plasma glucose levels.

The MDA level, sulfhydryl content, ferric ion reduction ability (FRA), and paraoxonase (PON)-like activity were quantified using the method described earlier [13]. Supplementary Method Section S3 describes the detailed methodology.

### 2.14. Hematoxylin and Eosin (H & E), Oil Red O (ORO), and Immunohistochemical (IHC) Staining

For histological analysis, tissues (liver and kidney) were sectioned using a cryo-microtome. First, tissue was embedded in Surgipath FSC22 frozen section solution (Leica, Nussloch, Germany). The tissue-embedded solution was immediately placed in the liquid nitrogen for rapid solidification (~5 min). The solidified tissue block was kept in a refrigerator (−21 °C) for 24 h, after which a tissue section (7 μm thick) was obtained using a cryo-microtome (Leica CM-1510S, Nussloch, Germany).

The histological changes in the liver and kidney were assessed by H&E staining, as previously described [36]. For ORO staining, the liver section (7 μm thick) was covered with ORO solution. After 5 min incubation at 60 °C, the section was washed with 60% isopropanol and analyzed under the microscope (Nikon, Tokyo, Japan).

The interleukin (IL)-6 in the liver section was determined by the IHC analysis. Tissue section (7 μm thick) was covered with the 200× diluted IL-6-specific antibodies (mouse IgG, ab9324, Abcam, Cambridge, UK). After overnight incubation (~16 h) at 4 °C in a moist environment, the section was washed with PBS (2 times) and subsequently added to 1000× diluted HRP-tagged secondary antibodies (anti-mouse IgG) as part of the EnVision HRP-polymer kit (K4001, Dako, Glostrup, Denmark). Finally, the IHC section was developed using chromogenic substrate (K4001, Dako, Glostrup, Denmark), and images were captured under the microscope (Nikon, Tokyo, Japan). The captured images were further processed to enhance the visibility and minimize the background by interchanging the IHC-stained brown color with red color using ImageJ software (https://imagej.net/ij, version 1.53, accessed on 6 June 2025) at a brown color threshold value (20–120).

### 2.15. Dihydroethidium (DHE) and Senescent-Associated-β-Galactosidase Staining

DHE staining of the tissue sections (liver and kidney) was performed according to the method described in Section 2.10.

For the detection of cellular senescence, the tissue section (7 μm thick) was covered with 0.1% solution of 5-bromo-4-choloro-3-indolyl-β-D-galactopyranoside solution (X-Gal). After 16 h of incubation at room temperature, the section was washed and visualized under a microscope (Nikon, Tokyo, Japan) to detect blue-stained senescent-positive cells.

### 2.16. Statistical Analysis

Statistical differences between groups (multiple comparisons) were assessed using the one-way analysis of variance (ANOVA) in SPSS software (version 29, Chicago, IL, USA). Data normality was assessed prior to performing the ANOVA. For the pairwise data comparison, a two-tailed *t*-test was conducted. The statistical analysis was performed using at least three independent experimental values per data point.

## 3. Results

### 3.1. HPLC Analysis of Medium Chain Fatty Acid (MCFA) Standard and Oil Samples

**Figure 2:**
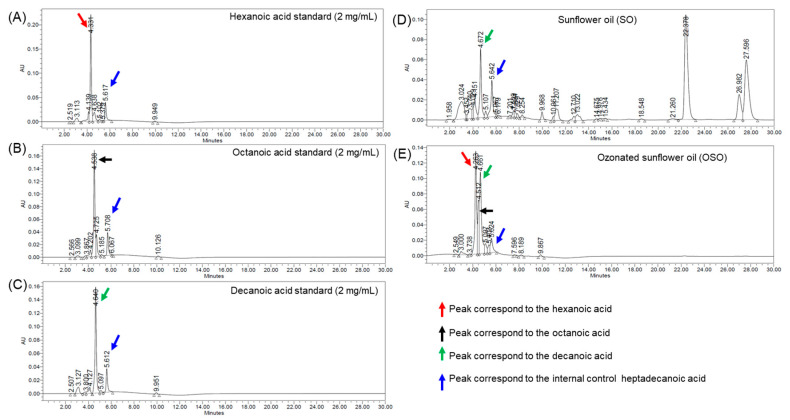
Representative HPLC chromatograms of standard (**A**) hexanoic acid, (**B**) octanoic acid, (**C**) decanoic acid, and extracted (**D**) sunflower oil (SO) and (**E**) ozonated sunflower oil (OSO). Red, black, and green arrows highlight the peaks corresponding to the hexanoic acid, octanoic acid, and decanoic acid, respectively, while blue arrows show the peak of the internal standard (heptadecanoic acid).

The HPLC method provided clear detection of the three MCFA markers and the internal standard within 6 min of analysis. As shown in Figure 2, hexanoic acid, octanoic acid, and decanoic acid were eluted at approximately 4.3, 4.5, and 4.6 min, respectively (Figure 2A–C). The peak of the internal standard, heptadecanoic acid, was shown at about 5.6 min. The peaks were practically separated, indicating that the chromatographic conditions were suitable for the simultaneous quantification of the analytes.

Comparison of chromatograms obtained from SO and OSO revealed notable differences in MCFA composition. In SO, a distinct peak corresponding to decanoic acid at a retention time (RT) of 4.6 min was observed, whereas peaks corresponding to hexanoic acid and octanoic acid were absent or below the detection limit. In contrast, the OSO chromatogram exhibited clearly detectable peaks corresponding to hexanoic acid at 4.3 min, octanoic acid at 4.5 min, and decanoic acid at 4.6 min, confirming the presence of all three MCFAs following ozonation.

Quantitative analysis and physicochemical properties, as shown in Table 1, revealed 1.23 mg/g of decanoic acid in SO, which increased to 2.88 mg/g in OSO, representing approximately a 2.3-fold increase following ozonation. Octanoic acid and hexanoic acid were not detected in SO under the HPLC analytical conditions. In contrast, OSO contained 1.73 mg/g of octanoic acid and 3.65 mg/g of hexanoic acid. These results indicate that ozonation of SO markedly enhances the formation or accumulation of MCFAs.

In addition, NPH derivatization of MCFAs and SO and OSO was performed prior to HPLC to enhance peak resolution and enable sensitive detection (Supplementary Figure S1). The standard hexanoic acid, octanoic acid and decanoic acid were eluted at RT 4.059 min, 6.369 min and 10.411 min, respectively. The HPLC chromatogram of OSO showed the presence of well-separated peaks corresponding to hexanoic acid (RT, 4.088 min), octanoic acid (RT, 6.216 min) and decanoic acid (RT, 10.404 min) in OSO. In the derivatized samples of SO, a minor peak of decanoic acid was detected, while the characteristic peaks of octanoic acid and hexanoic acid were not detected. Also, the quantification of MCFAs following NPH derivatization is comparable to that of the non-derivatized samples, as shown in Table 1.

### 3.2. Identification of Medium Chain Fatty Acids in Oil Samples by LC/MS Analysis

The HPLC results showing the presence of decanoic acid, octanoic acid, and hexanoic acid (SO vs. OSO) were further validated by LC/MS analysis. For the scan-mode data, MS/MS fragmentation was carried out, and collision energy values were applied to detect fatty acid signals such as decanoic acid, octanoic acid or hexanoic acid using standard solutions of each type of fatty acid.

**Figure 3:**
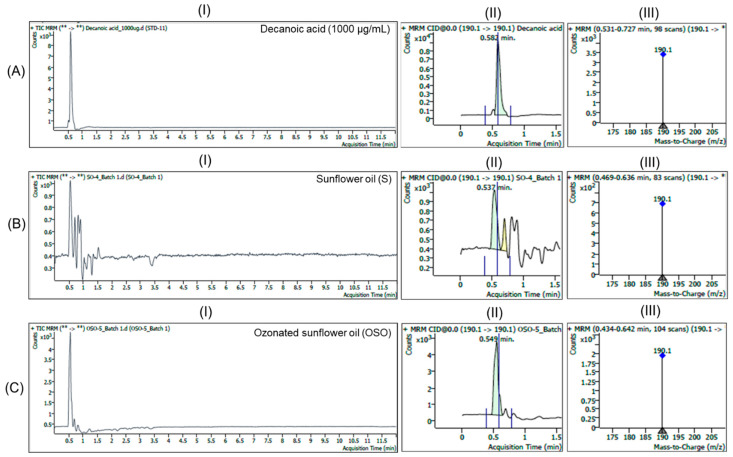
LC/MS multiple reaction monitoring (MRM) mode total ion chromatograms and mass spectra of (**A**) standard decanoic acid, (**B**) sunflower oil (SO), and (**C**) ozonated sunflower oil (OSO). (**I**–**III**) represent the total ion chromatogram, a magnified view of the peak corresponds to decanoic acid, and the mass spectra (*m*/*z* 170–200) of the respective images.

Based on the fragmentations, the LC/MS operating in multiple reaction monitoring (MRM) mode under the positive ESI condition (Figure 3) for standard decanoic acid (1000 µg/mL in acetonitrile/methanol (1:1, *v*/*v*) with 0.1% formic acid) produced a distinct peak at 0.582 min as RT, corresponding to a mass spectrum that displayed a dominant ion at *m*/*z* 190.1 [M + NH_4_]^+^, suggesting compound identity. In SO, a distinct peak was detected at 0.537 min under the same MRM transition. The MS spectrum acquired at this retention window showed the characteristic ion at *m*/*z* 190.1, confirming the presence of decanoic acid in SO. In OSO, a well-defined peak was observed at 0.549 min with *m*/*z* 190.1, confirming the identity of decanoic acid in OSO. The higher signal observed in OSO compared with SO suggests that ozonation may enhance decanoic acid formation in SO. Overall, the LC/MS analysis confirmed the detection of decanoic acid in both SO and OSO, based on peak specificity at the RT of standard decanoic acid, with a markedly higher abundance in the ozonated sample.

**Figure 4:**
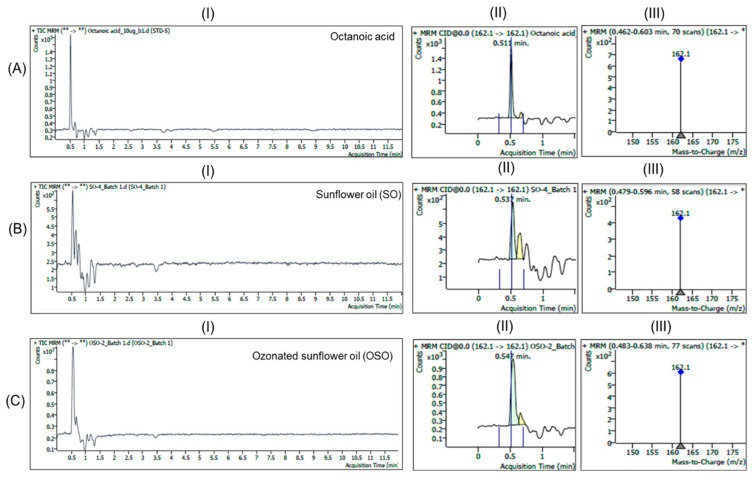
LC/MS multiple reaction monitoring (MRM) mode total ion chromatograms and mass spectra of (**A**) standard octanoic acid, (**B**) sunflower oil (SO), and (**C**) ozonated sunflower oil (OSO). (**I**–**III**) represent the total ion chromatogram, a magnified view of the peak corresponds to octanoic acid and the mass spectra (*m*/*z* 160–200) of the respective images.

As in the analysis of decanoic acid, the multiple reaction monitoring (MRM) mode was used for LC/MS analysis of standard octanoic acid or hexanoic acid. In the chromatograms of MRM mode under the positive ESI condition (Figure 4), the distinct peaks at an RT of 0.511 min corresponded to mass spectrums that displayed a dominant ion at *m*/*z* 162.1 [M + NH_4_]^+^, suggesting compound identity of octanoic acid. The MS spectrum of the SO sample acquired at this retention window only showed the ion at *m*/*z* 162.1 with the signal (peak area) comparable to the background signal of zero concentration in solvent, confirming the near absence of octanoic acid in SO. In OSO, a well-defined peak was observed at 0.547 min with *m*/*z* 162.1, confirming the identity of octanoic acid in OSO. The apparent signal of octanoic acid observed in OSO compared with the little signal of octanoic acid in SO indicates that ozonation might enhance the formation of octanoic acid in sunflower oil.

**Figure 5:**
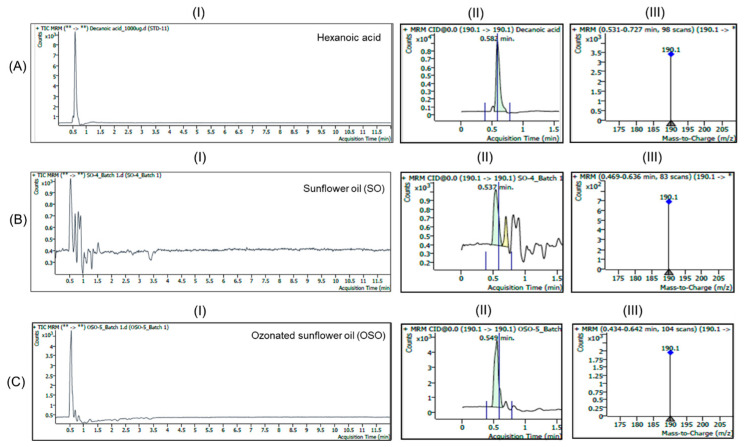
LC/MS multiple reaction monitoring (MRM) mode total ion chromatograms and mass spectra of (**A**) standard hexanoic acid, (**B**) sunflower oil (SO), and (**C**) ozonated sunflower oil (OSO). (**I**–**III**) represent the total ion chromatogram, a magnified view of the peak corresponds to hexanoic acid and the mass spectra (*m*/*z* 160–200) of the respective images.

In the chromatograms of MRM mode under the negative ESI condition (Figure 5), the distinct peaks at an RT of 0.691 min corresponded to mass spectrums that displayed a dominant ion at *m*/*z* 161.1 [M + HCOO]^−^, suggesting compound identity of hexanoic acid. The MS spectrum of the SO sample acquired at this retention window only showed the ion at *m*/*z* 161.1 with the signal similar to the background signal of zero concentration in solvent, confirming the near absence of hexanoic acid in SO. In OSO, a well-defined peak was observed at 0.658 min with *m*/*z* 161.1, confirming the presence of hexanoic acid in OSO. The apparent signal of hexanoic acid observed in OSO, compared with the low signal in SO, suggests that ozonation may enhance its formation in sunflower oil.

Based on the results above, the LC/MS analysis confirmed the detection of octanoic acid and hexanoic acid in OSO, based on peak specificity at the retention times of the standards, with distinct abundance in the ozonated sample compared to almost absent levels of the two fatty acid species in SO.

### 3.3. In Vitro Antioxidant Abilities and Inhibition of LDL Oxidation by MCFAs

**Figure 6:**
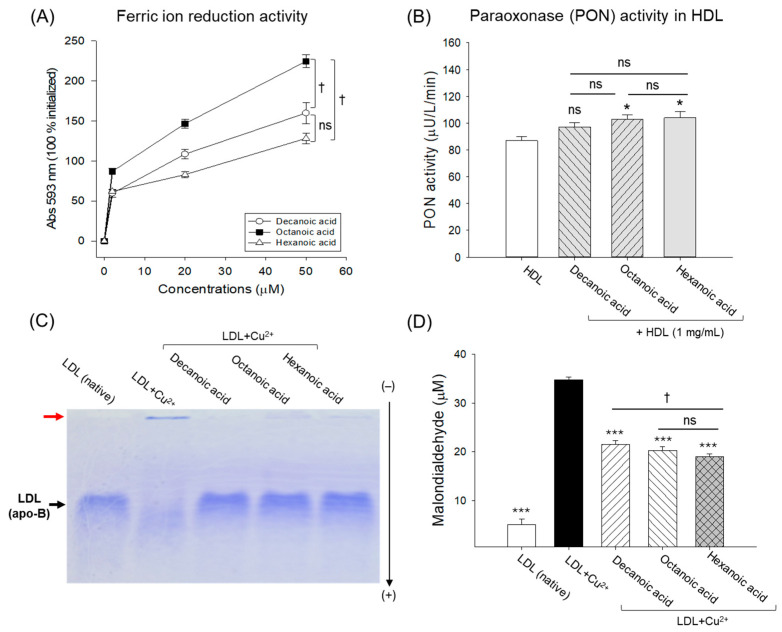
Comparative antioxidant and low-density lipoprotein (LDL) protective effect of decanoic acid, octanoic acid, and hexanoic acid (at 50 μM concentration). (**A**) Ferric ion reduction activity; ^†^ (*p* < 0.05) highlights the statistical difference between the marked groups (FRA). (**B**) High-density lipoprotein (HDL) associated paraoxonase (PON) activity; * (*p* < 0.05) highlights the statistical difference vs. the HDL group. (**C**) Electrophoretic mobility of LDL (apo-B fraction) exposed to CuSO_4_. The red arrow indicates the gel-loading front. (**D**) Quantification of malondialdehyde (MDA) as a lipid peroxidation marker in the LDL exposed to CuSO_4_; *** (*p* < 0.001) highlights the statistical difference vs. the LDL + Cu^2+^ group, ^†^ (*p* < 0.05) highlights the statistical difference between the marked groups. The “ns” depicts a nonsignificant difference.

As shown in Figure 6A, all three MCFAs displayed a dose-dependent effect on ferric ion reduction ability. However, octanoic acid exhibited the highest FRA at all the tested concentrations (5–50 μM). At a 50 μM concentration, the FRA of octanoic acid was significantly 1.4-fold and 1.7-fold higher than the FRA detected in decanoic acid and hexanoic acid, respectively. No significant difference between the FRA of decanoic acid and hexanoic acid was noticed at the 50 μM concentration.

Compared to the PON activity detected in the native HDL, the HDL treated with octanoic acid and hexanoic acid showed a ~1.2-fold (*p* < 0.05) higher PON activity (Figure 6B). PON activity of decanoic acid-treated HDL also showed a minor enhancement compared to that of native HDL; however, the difference was not statistically significant.

As depicted in Figure 6C, a faint and smeared LDL band (corresponding to LDL oxidation) with high aggregation in the loading well appeared in the LDL treated with CuSO_4_. In contrast, the native LDL (untreated) showed sharp banding without aggregation in the loading well. Treatment with MCFAs substantially protected LDL from oxidation, as reflected by a sharp band intensity without smearing or aggregation in the well. Likewise, a high lipid peroxidation (quantified as MDA levels) was observed in the LDL + CuSO_4_-treated group, which was significantly 6.8-fold higher than that in native LDL (Figure 6D). The CuSO_4_-induced lipid peroxidation in LDL was significantly reduced by ~1.8-fold (*p* < 0.001) with MCFA treatment.

The finding establishes the antioxidant activity of MCFAs, their positive impact on HDL-associated PON activity, and their ability to prevent LDL oxidation.

### 3.4. Survivability of Embryos

**Figure 7:**
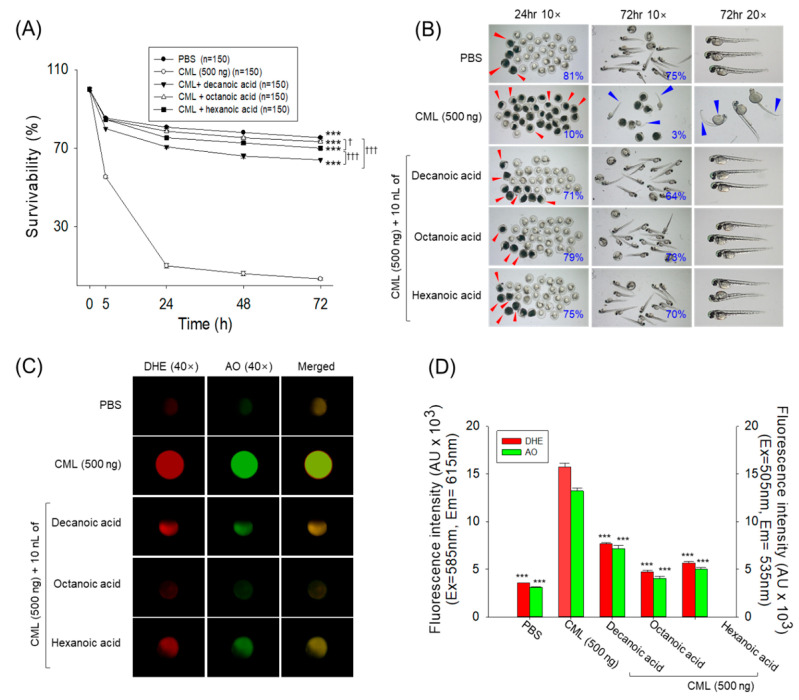
Comparative effect of decanoic acid, octanoic acid, and hexanoic acid microinjection (final 2 ng) on zebrafish embryos (*n* = 150). (**A**) Survival kinetics during 72 h post-treatment. (**B**) Pictorial representation of embryos. The percentage of surviving embryos is mentioned in the blue numbers. Red and blue arrows depict the death and developmental deformities in embryos, respectively. (**C**) Dihydroethidium (DHE) and acridine orange (AO) fluorescent staining of the embryos. (**D**) Quantification of DHE and AO fluorescent intensities. PBS and CML are abbreviated for phosphate-buffered saline and carboxymethyllysine, respectively. The *** (*p* < 0.001) underscores the statistical difference between the groups compared to the CML-injected group. ^†^ (*p* < 0.05), and ^†††^ (*p* < 0.001) underscore the statistical difference between the marked groups.

Embryo survivability consistently decreased in a time-dependent manner in the CML-treated group (Figure 7A). At 5 h post-treatment with CML, the survivability of embryos reached 55%, which further reached 10% at 24 h and finally reached 3% at 72 h post-treatment. In contrast to this, the PBS-treated control group displayed 75% embryo survivability at 72 h post-treatment, which was 25-fold higher than the embryo survivability in the CML-treated group. Cotreatment of all three MCFAs (2 mM) with CML displayed a substantial effect on the prevention of CML-induced embryo death. Among the MCFAs, octanoic acid showed a substantially higher effect with 73% survivability, followed by hexanoic acid (70%) and decanoic acid (64%) at 72 h post-treatment.

The morphology of the embryos depicted in Figure 7B showed that most surviving embryos in the CML-treated group exhibited developmental defects, including tail-fin curvature, yolk-sac edema, and back bending. In contrast, most embryos from all three MCFA-treated groups showed normal developmental morphology, very similar to that observed in the PBS control group.

The highest DHE and AO fluorescence levels (indicative of ROS generation and apoptosis) were observed in CML-treated embryos, which were 4.4-fold and 4.2-fold higher than the respective levels in the PBS-treated control group (Figure 7C,D). The treatment of decanoic acid, octanoic acid, and hexanoic acid significantly (*p* < 0.001) reduced the CML-triggered ROS generation and apoptosis in embryos.

The findings indicated that all three MCFAs have an inhibitory effect against CML, inducing ROS generation, consequently protecting embryos from acute apoptotic death.

### 3.5. Survivability and Swimming Activity of Adult Zebrafish

**Figure 8:**
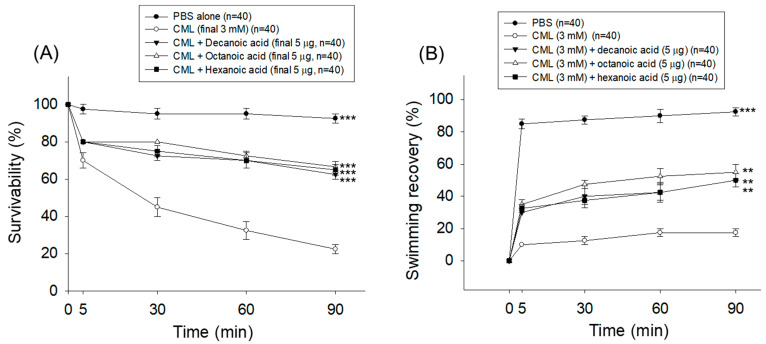

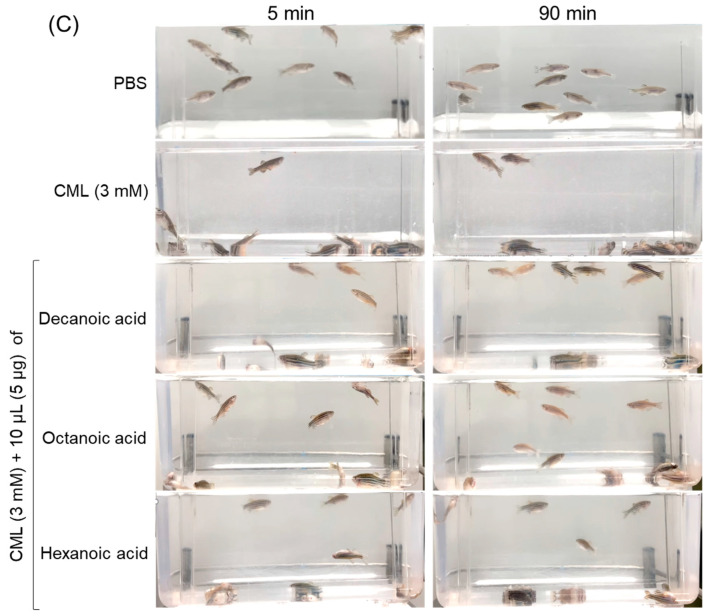
Comparative effect of decanoic acid, octanoic acid, and hexanoic acid treatment (5 μg) on adult zebrafish (*n* = 40) (**A**) survivability and (**B**) swimming recovery during 90 min post-treatment. (**C**) Still images of the zebrafish captured at 5 min and 90 min post-treatment. PBS and CML are abbreviated for phosphate-buffered saline and carboxymethyllysine, respectively. ** (*p* < 0.01) and *** (*p* < 0.001) underscore the statistical difference compared to the CML-injected group.

As shown in Figure 8A, the CML-treated group showed acute death and the lowest survivability (22%) at 90 min post-treatment; in contrast, the PBS-treated control group showed the highest survivability (93%). Treatment of all three MCFAs showed a substantial counter effect against CML-posed mortality, reflected by around 63~67% survivability, which was ~2.7-fold (*p* < 0.001) higher than the survivability observed in the CML-treated group at 90 min post-treatment.

Consistently, the lowest recovery of swimming ability, which was merely 17%, was observed in the CML-injected group at 90 min post-treatment, whereas the PBS control group showed the highest (93%) swimming recovery (Figure 8B). The cotreatment of three MCFAs (decanoic acid, octanoic acid, and hexanoic acid) with CML effectively improved the CML-compromised swimming activity manifested by 50~55% swimming recovery, which was notably ~3-fold higher than the swimming activity of the CML-treated group at 90 min post-treatment.

Interestingly, all three MCFAs displayed a statistically similar protective effect against CML-triggered zebrafish death and swimming impairments.

### 3.6. Plasma Lipid and Glucose Level

**Figure 9:**
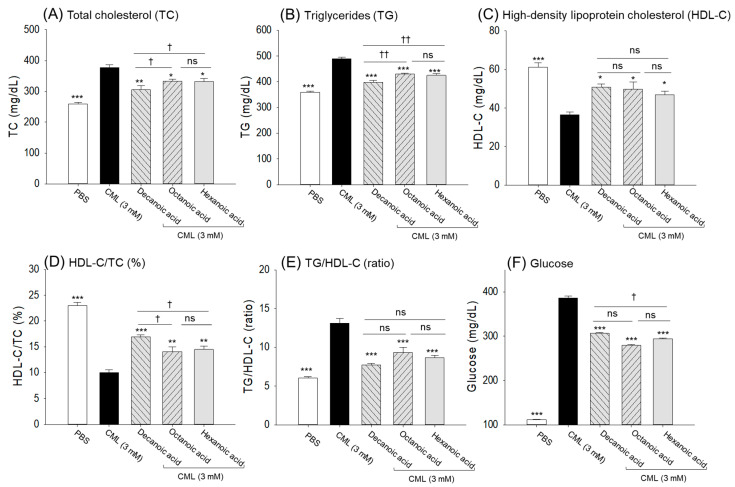
Comparative effect of decanoic acid, octanoic acid, and hexanoic acid treatment (5 μg) on the blood lipoprotein profile (**A**–**E**) and glucose (**F**) levels of adult zebrafish. PBS and CML are abbreviated for phosphate-buffered saline and carboxymethyllysine, respectively. The data point represents the mean ± SEM from the four (*n* = 4) independent experiments. * (*p* < 0.05), ** (*p* < 0.01), and *** (*p* < 0.001) underscore the statistical difference between the groups compared to the CML-injected group. ^†^ (*p* < 0.05), ^††^ (*p* < 0.01) represent the statistical difference between the groups. “ns” represents the nonsignificant difference.

The CML-injected group showed the highest plasma TC (377.2 ± 9.8 mg/dL) and TG (489.1 ± 5.9 mg/dL) levels that were 1.3-fold and 1.4-fold higher than their respective levels detected in the PBS control group (Figure 9A,B). Treatment with decanoic acid, octanoic acid, and hexanoic acid effectively reduced CML-elevated TC levels to 305.5 mg/dL, 333.7 mg/dL, and 332.6 mg/dL, and TG levels to 397.6 mg/dL, 429.5 mg/dL, and 414.6 mg/dL, respectively. However, among the three MCFAs, decanoic acid proved most effective compared to octanoic and hexanoic acids to reduce the TC and TG levels.

The minimum HDL-C level was detected in the CML-treated group, which was 1.7-fold lower than the HDL-C level detected in the PBS control group (Figure 9C). The CML-diminished HDL-C level was ~1.4-fold elevated as a result of MCFA treatment. Interestingly, all the MCFAs (decanoic, octanoic, and hexanoic) displayed a statistically similar effect to elevating the CML-diminished HDL-C level.

Consistently, the CML-treated group showed the lowest HDL-C/TC (%), which was substantially elevated (~1.5-fold) by exposure to MCFAs (Figure 9D). Among the tested MCFAs, decanoic acid showed ~15% greater efficacy than octanoic and hexanoic acids in increasing HDL-C/TC (%). Also, treatment with MCFAs substantially minimizes the CML-elevated TG/HDL-C ratio (Figure 9E).

The maximum blood glucose level was observed in the CML-treated group (386.6 ± 4.1 mg/dL), which was significantly reduced to ~1.3-, 1.4-, and 1.4-fold by treatment with decanoic, octanoic, and hexanoic acids (Figure 9F). Compared with decanoic acid and hexanoic acid, octanoic acid produced an ~8% greater effect in lowering CML-elevated blood glucose levels.

### 3.7. Plasma Status of Oxidative Stress and Antioxidant Markers

**Figure 10:**
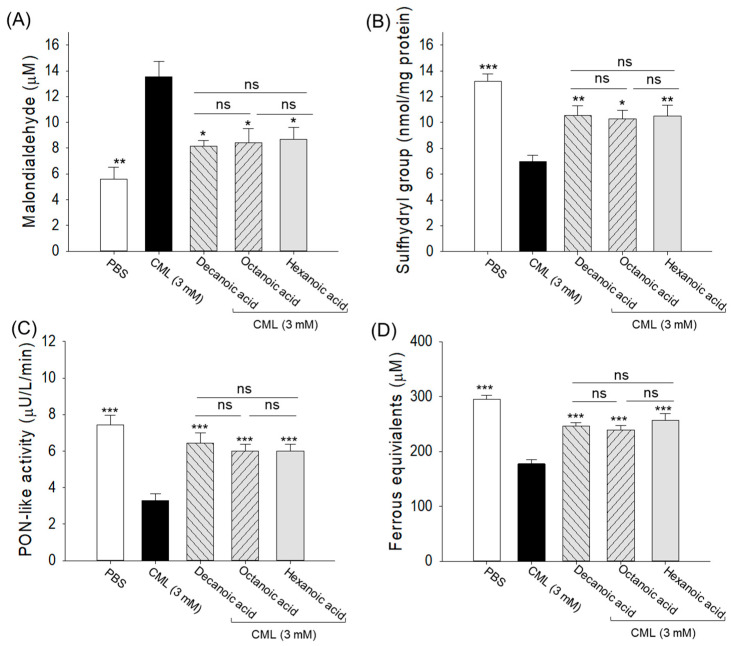
Comparative (**A**) malondialdehyde level, (**B**) sulfhydryl content, (**C**) paraoxonase (PON)-like activity, and (**D**) ferric ion reduction ability (FRA) in the blood of decanoic acid-, octanoic acid-, and hexanoic acid-treated (5 μg) zebrafish. PBS and CML are abbreviated for phosphate-buffered saline and carboxymethyllysine, respectively. Each value in the bar graph represents the mean ± SEM of four (*n* = 4) independent experiments. The * (*p* < 0.05), ** (*p* < 0.01), and *** (*p* < 0.001) depict the statistical difference compared to the CML-injected group. “ns” represents the nonsignificant difference.

A 2.4-fold higher plasma MDA level was detected in the CML-treated group than the basal MDA level detected in the PBS control group (Figure 10A). Treatment with the MCFAs minimizes the CML-elevated MDA level by ~1.6-fold. Unlike the MDA, a 1.9-fold reduction in plasma sulfhydryl content was observed in the CML-treated group relative to the PBS control group (Figure 10B). The MCFA treatment elevated the sulfhydryl content by ~1.5-fold (*p* < 0.001) relative to the CML-treated group.

The plasma FRA and PON-like activities were 1.6-fold and 2.2-fold reduced by the exposure to CML compared to the PBS-treated group (Figure 10C,D). Overall, ~1.4-fold and ~1.8-fold higher FRA and PON-like activities were quantified in the MCFA-treated group than in the CML-treated group.

The findings demonstrate that MCFAs play a significant role in mitigating oxidative stress and improving antioxidant capacity. Remarkably, all the MCFAs (decanoic acid, octanoic acid, and hexanoic acid) exhibited statistically comparable effects in alleviating CML-induced oxidative stress and restoring impaired antioxidant defenses.

### 3.8. Plasma AST and ALT Level

**Figure 11:**
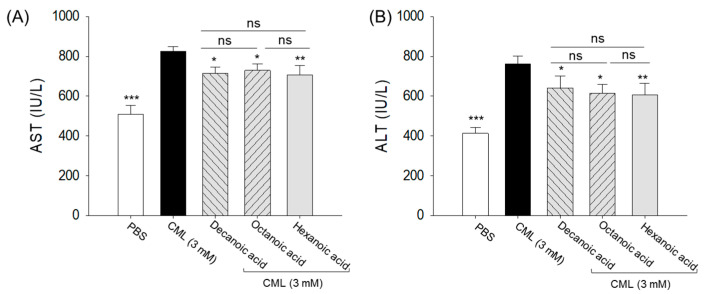
A comparative study of blood hepatic function biomarker levels (**A**) aspartate aminotransferase (AST) and (**B**) alanine aminotransferase (ALT) in the zebrafish treated with hexanoic acid, octanoic acid, and decanoic acid. PBS and CML are abbreviated for phosphate-buffered saline and carboxymethyllysine, respectively. * (*p* < 0.05), ** (*p* < 0.01) and *** (*p* < 0.001) represent the statistical difference compared to the CML-injected group. The “ns” depicts a nonsignificant difference.

Compared to the PBS control group, significantly elevated 1.6-fold and 1.8-fold AST and ALT levels were observed in the CML-treated group, respectively (Figure 11). The CML-elevated AST and ALT levels were reduced by ~1.2-fold with MCFA treatment. All MCFAs produced comparable reductions in AST and ALT levels, with no significant differences observed among decanoic acid, octanoic acid, and hexanoic acid.

### 3.9. Hepatic Tissue Analysis

**Figure 12:**
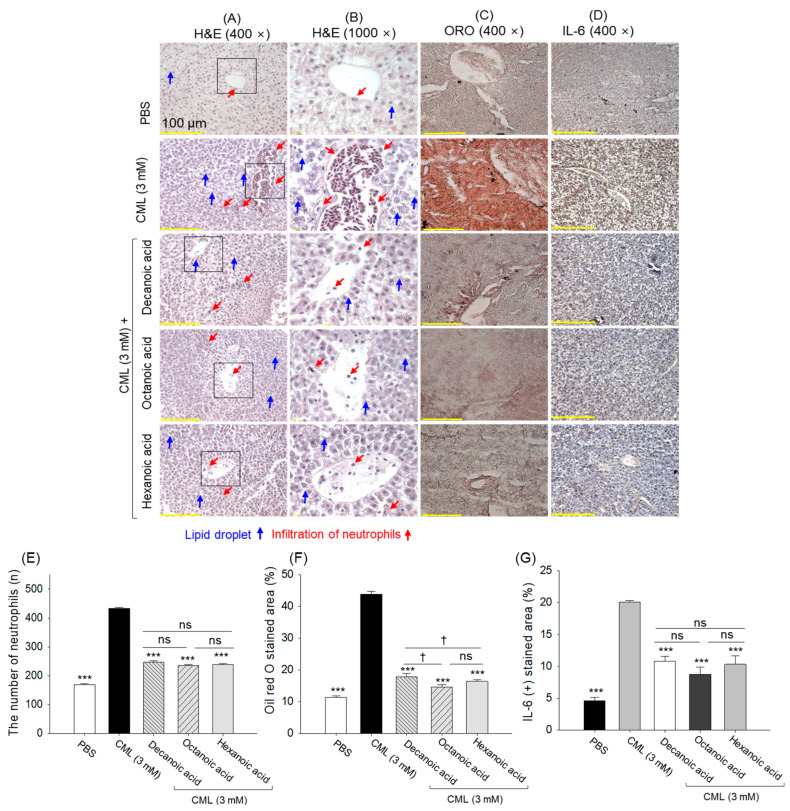
Comparative liver histological analysis of decanoic acid-, octanoic acid-, and hexanoic acid-treated zebrafish. (**A**) Hematoxylin and eosin (H & E) staining at 400× magnification. (**B**) H&E-stained images (1000× magnified) from the section covered inside the black box of the respective images in subset A. (**C**) Oil red O (ORO) staining. (**D**) Interleukin (IL)-6 staining was performed by immunohistochemistry (IHC). Quantification of (**E**) neutrophil counts, (**F**) ORO-stained, and (**G**) IL-6-stained area, respectively. PBS and CML are abbreviated for phosphate-buffered saline and carboxymethyllysine, respectively. *** (*p* < 0.001) depict the statistical difference compared to the CML-treated group. ^†^ (*p* < 0.05) underscore statistical significance between the marked groups. The “ns” depicts a nonsignificant difference.

The H&E staining revealed a 2.5-fold higher neutrophil count in the liver of the CML-treated group compared to the PBS control group (Figure 12A,B,E). Overall, a ~1.8-fold reduced neutrophil count was detected in the MCFA-treated group compared with the CML-treated group. Likewise, high lipid accumulation, reflected by a 3.9-fold higher ORO-stained area than the PBS control group, was observed in the CML-treated group (Figure 12C,F). The treatment with MCFA effectively reduced the CML-associated elevated hepatic lipid accumulation. Contrary to decanoic acid, treatment with octanoic acid and hexanoic acid demonstrated ~14% higher efficiency in inhibiting CML-triggered lipid accumulation.

The IHC staining revealed a massive IL-6 production in the hepatic tissue treated with CML, reflected by the highest IL-6-stained area (20.2%) (Figure 12D,G). Treatment with MCFAs substantially reduced IL-6 production as indicated by an IL-6-stained area of only 8.8–10.8%, which is 2.3~1.8-fold lower than the IL-6-stained area that appeared in the CML-treated group. The findings suggest that MCFAs effectively counter CML-triggered inflammation by reducing neutrophil infiltration, IL-6 generation, and alleviating fatty liver alterations.

### 3.10. ROS Generation and Senescence in Liver

**Figure 13:**
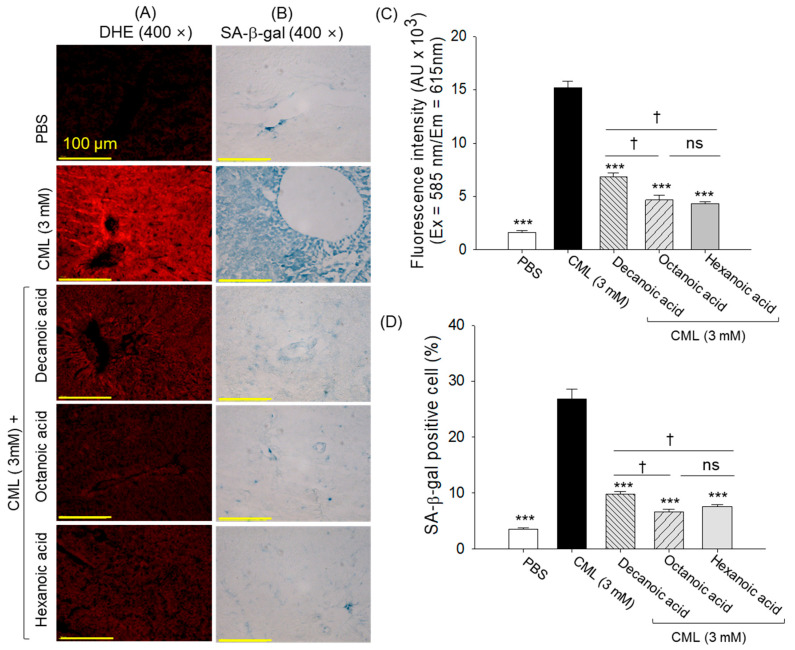
Comparative effect of decanoic acid, octanoic acid, and hexanoic acid treatment (5 μg) on zebrafish liver reactive oxygen species (ROS) and senescence detected by (**A**) dihydroethidium staining (DHE) and (**B**) senescent-associated β-galactosidase (SA-β-gal) assay. Quantification of (**C**) DHE fluorescent intensity and (**D**) SA-β-gal-positive cells. PBS and CML are abbreviated for phosphate-buffered saline and carboxymethyllysine, respectively. *** (*p* < 0.001) highlights the statistical significance between the groups with respect to the CML-injected group. ^†^ (*p* < 0.05) underscore the statistical difference between the marked groups. The “ns” depicts a nonsignificant difference.

DHE fluorescent staining and senescent-associated β-gal staining revealed 9.4-fold and 7.6-fold higher ROS generation and senescence in the liver of the CML-injected group compared to the PBS control group (Figure 13A–D). Treatment with MCFAs showed a substantial counter-effect against CML-triggered ROS generation and cellular senescence, as evidenced by a 2.2~3.5-fold and 2.7~4.1-fold reduction in DHE fluorescence intensity and in senescent-positive cells compared with the CML-treated group. Compared with decanoic acid, treatment with octanoic acid and hexanoic acid showed ~30% and ~23% reduced DHE fluorescence and senescence, respectively.

### 3.11. Renal Tissue Analysis

**Figure 14:**
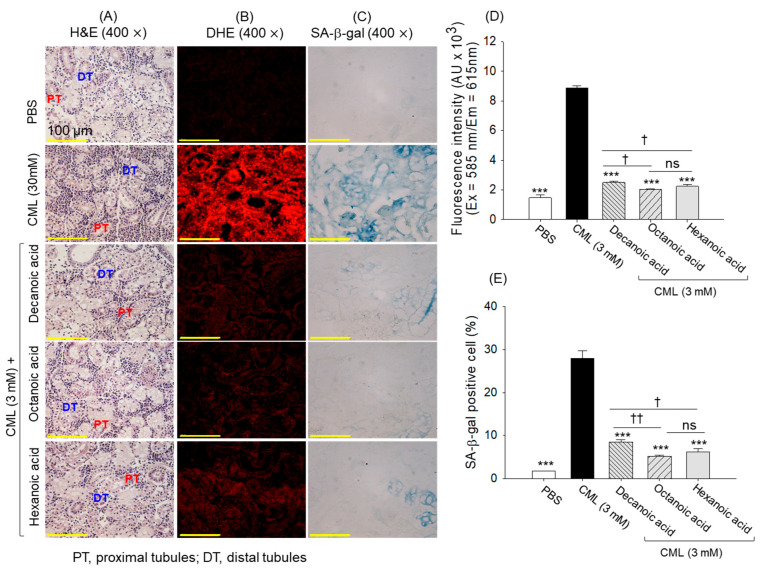
Comparative effect of decanoic acid, octanoic acid, and hexanoic acid treatment (5 μg) on the kidney histology of zebrafish. (**A**) Hematoxylin and eosin (H&E) staining; PT and DT are abbreviated for proximal and distal tubules, respectively. (**B**) Dihydroethidium (DHE) fluorescent staining and (**C**) senescent-associated β-galactosidase (SA-β-gal) staining. Quantification of (**D**) DHE fluorescent intensity and (**E**) SA-β-gal-positive cells. PBS and CML are abbreviated for phosphate-buffered saline and carboxymethyllysine, respectively. *** (*p* < 0.001) underscores the statistical difference between the groups compared to the CML-treated group. ^†^ (*p* < 0.05), ^††^ (*p* < 0.01) highlight the statistical relevance between the marked groups.

Well-organized distal (DT) and proximal tubular (PT) structures were noticed in the PBS-injected group that were substantially altered by exposure to CML. In the CML-treated group, sparsely populated DT and PT structures with dilated tubular lumens and cellular debris were observed (Figure 14A). Treatment with MCFAs substantially protects the kidney from CML-induced damage; however, minor dilated tubular lumens and cellular debris were observed at certain positions.

DHE staining revealed substantial ROS generation in the CML-injected group, which accounts for a 5.9-fold higher ROS level than the basal level detected in the PBS-injected group (Figure 14B,D). MCFA-treated groups showed a 3.5~4.3-fold lower ROS generation compared to the CML group, attesting to the MCFAs efficacy to inhibit CML-induced ROS generation. Similarly, a 15.5-fold higher prevalence of senescent-positive cells than in the PBS control group was observed in the CML-injected group. The CML-induced cellular senescence was substantially inhibited by treatment with MCFAs, as reflected by a 3.3~5.2-fold lower number of senescent positive cells in the MCFA-treated groups compared to the CML-injected group (Figure 14C,E). Importantly, relative to decanoic acid, octanoic and hexanoic acids resulted in ~30% and ~16 decreases in senescence and ROS production.

## 4. Discussion

During ozonation, ozone gas flushes into the oil, where it is trapped at the unsaturation site via electrophilic addition, leading to the formation of several products, such as hydroperoxides, Criegee ozonide, and carboxylic acids [40,41], which impart a distinct odor and pharmacological properties to the ozone oil. As far as our knowledge, the current study is the first report to compare MCFAs in SO before and after ozonation, revealing that ozonation significantly increased hexanoic acid, octanoic acid and decanoic acid. Notably, OSO exclusively contained hexanoic and octanoic acids and had a much higher decanoic acid level than the non-ozonated oil. Traditionally, gas chromatography (GC) has been recognized as an ideal method for detecting fatty acids. However, with the development of sensitive HPLC methods and the use of a variety of derivatization approaches, HPLC demonstrated broader applicability for the detection of fatty acids in diverse biological matrices. The successful use of HPLC has been employed in many studies to analyze short and MCFAs in food and biological samples [42,43].

The strong rancid odor of OSO is likely attributed to high concentration of decanoic acid along with exclusive presence of octanoic and hexanoic acids, as these MCFAs exhibit exceptionally low odor threshold values (0.001–0.008 ppm) and high volatility, reflected in their low melting point range −3.4 to 31.5 °C (Table 1). The study findings suggest that ozonation not only impacts the formation of a variety of ozone-catalyzed compounds but also impacts the formation of MCFAs. Our previous reports suggested a functional superiority of OSO over SO [12,14,44]. The typical presence of a variety of carboxylic acids, hydroperoxides, ozonides and other peroxide species [17] in OSO is responsible for its functionality. However, among the several contributing factors, the distinct presence of MCFAs in OSO may be one of the reasons underlying its higher functionality. The assumption is in accordance with earlier reports documenting the significant role of MCFAs in health and disease through the regulation of diverse cellular processes [45,46]. In particular, the beneficial effects of MCFAs on skin health, weight management, immunomodulation and cardiovascular function have been documented [45]. In the present study, a comparative evaluation of the functional properties of individual MCFAs (decanoic acid, octanoic acid, and hexanoic acid) was performed using both in vitro assays and animal studies. The in vitro assays suggest that all the MCFAs have substantial antioxidant activity, augment HDL-associated antioxidant PON activity and protect LDL from oxidative damage. The free radical scavenging and FRAP activities of decanoic acid have been reported [47], which align with the present findings. However, to the best of our knowledge, no report has documented the effects of MCFAs on PON activity and on the prevention of LDL oxidative damage. The present results also justify the previous findings, deciphering OSO’s substantial FRA ability and impact on the activity enhancement of PON [10]. It can be postulated that the presence of MCFAs in OSO is one of the contributing factors responsible for the enhancement of PON activity, as reported in our previous study [10].

CML is well known to induce oxidative stress and inflammation [48,49,50]. In line with this, CML-treated embryos exhibited high oxidative stress and apoptotic death, which were effectively countered by exposure to MCFAs. This protective effect is attributed to the antioxidant properties of MCFAs, which mitigate ROS generation and subsequent apoptosis, consistent with reports linking antioxidants to the inhibition of ROS-induced apoptosis [51]. Corroborating our previous findings, which demonstrated the cytoprotective effect of OSO against oxidative stress [10], the present findings suggest that the presence of MCFAs in OSO is one of the contributing factors to its cytoprotective effects, due to their substantial antioxidant properties. In adult zebrafish, treatment with MCFAs alleviated severe swimming impairment and acute mortality induced by CML. This protective effect is attributed to the antioxidant and anti-inflammatory properties of MCFAs, given that CML-induced oxidative stress and inflammation are key drivers of these adverse outcomes [52]. Nevertheless, further mechanistic studies are required to elucidate the precise mechanism by which MVFAs confer this protection against CML-induced acute death and swimming impairment.

The CML impact on the induction of oxidative stress and impairment of antioxidants has been recognized [52]. Likewise, an elevation in MDA levels, a decrease in sulfhydryl content, and compromised FRA and PON-like activities were observed in the blood of the CML-treated group. Sulfhydryl group is considered an important antioxidant that neutralizes the peroxyl radicals [53,54] and its diminished level is associated with compromised antioxidant status and inflammatory disorders [55,56]. Plasma FRA activity reflects the total antioxidant capacity of the blood, and a higher value indicates a stronger antioxidant defense to mitigate oxidative stress [57]. Likewise, PON is an important antioxidant associated with HDL, has an inhibitory impact on lipid peroxidation [58], and its reduced activity is linked with myocardial infarction and liver disease [59,60]. The CML-induced elevated MDA level and diminished plasma sulfhydryl, FRA, and PON-like activities, which are Ca^2+^-dependent, were significantly reversed by MCFAs, attesting to their substantial effect in curtailing oxidative stress by elevating the antioxidant defense system. These findings align with the outcomes of the in vitro antioxidant assay, which demonstrated the free radical scavenging activity of MCFAs and their role as PON activators. The in vivo protective effect is likely attributed to multiple complementary mechanisms. In particular, the direct ROS-scavenging properties of MCFAs appear to play a substantial role, together with their ability to enhance PON and other antioxidant defenses, thereby effectively mitigating the harmful effects of oxidative stress. Notably, in contrast to the in vitro assay, where octanoic acid exhibited significantly higher FRA than hexanoic acid and decanoic acid, no significant difference in in vivo FRA activity was observed between these MCFAs. This discrepancy is probably influenced by physiological factors such as absorption, metabolism, tissue distribution and cellular antioxidant systems, which can minimize the difference among the MCFAs. The current findings are supported by existing literature showing that decanoic acid suppresses oxidative stress, as evidenced by reduced malondialdehyde (MDA) levels, and potentiates the cellular antioxidant system by modulating superoxide dismutase (SOD) and glutathione (GSH) [61] activity. Likewise, octanoic acids have been shown to reduce oxidative stress, and enhancement of antioxidant activity has been recognized [62].

High oxidative stress and inflammation in the CML-treated group are linked to a disturbed plasma lipid profile, as it has been well established that inflammation induces metabolic diseases [63] and dyslipidemia [64]. Elevated proinflammatory cytokines, mainly TNF-α and IL-6, are inversely associated with blood HDL-C levels and HDL functionality, including PON activity, demonstrating a notable adverse effect of inflammation on HDL. Due to the substantial anti-inflammatory effect of MCFAs, it balances the CML-disturbed lipid profile. The findings are consistent with earlier reports deciphering the impact of octanoic acid and decanoic acid on lipid metabolism [65,66,67]. Similarly, hexanoic supplementation was effective in reducing plasma TC and TG levels elevated by a high-fat diet in mice [66]. Also, reports described the effect of oral consumption with MCFAs on elevating HDL-C levels, indirectly supporting the present outcomes [68]. The current findings align with our previous results, in which OSO supplementation reduced TC and TG while elevating HDL-C/TC (%), underscoring the role of MCFAs in OSO as one of the modulating factors of plasma lipid metabolism and corroborating the present findings. A high blood glucose level induced by CML was substantially mitigated by exposure to MCFAs. The results aligned with the literature show that hexanoic acid inhibits high-fat diet-induced hyperglycemia [66]. Additionally, dietary MCFAs are known to improve insulin resistance [45,66,69], supporting their role in blood glucose management and strengthening these findings.

CML is known to damage the liver by inducing inflammation [70]. Similarly, hepatic inflammation, characterized by elevated neutrophil counts, increased IL-6 production, and steatosis, was observed in the CML-treated group and was effectively reversed by the MCFAs. In addition to the histological changes, decreased AST and ALT levels, which are important plasma hepatic function biomarkers [71], were detected in the MCFAs-treated groups, attesting to their hepatoprotective role. The findings are in good agreement with the previous reports deciphering the impact of MCFAs to manage hepatic steatosis [45,72,73] and on the reduction of inflammatory cytokines, including TNF-α, IL-1β, and IL-6, thus documenting a substantial anti-inflammatory and liver-protective effect [61]. Besides its impact on steatosis and inflammation, MCFA treatment effectively minimizes CML-induced ROS generation and hepatic cellular senescence. The antioxidant effect of MCFAs was the primary reason for the inhibition of ROS generation. Also, lower senescence is associated with lower ROS production in the MCFA-treated groups, as a direct association between oxidative stress and senescence has been documented [74]. Our previous reports elucidated the OSO hepatoprotective role [12,14], and the current findings add value by indicating that the presence of MCFAs in OSO is one of the contributing factors to its hepatoprotective effect.

A negative effect of ROS on kidney health has been described [75]. Herein, poor kidney health in the CML-injected group, in response to high ROS production, was observed and was significantly mitigated by exposure to MCFAs. The antioxidant properties of MCFAs play a central role in reducing ROS levels and, consequently, protecting the kidney. In addition to their antioxidant nature, a substantial impact of MCFAs on the enhancement of HDL level and functionality is another possible reason for the kidney protective role. This notion is consistent with earlier reports suggesting that HDL has a positive impact on kidney health [76]. Moreover, the higher sulfhydryl content in the MCFA-treated groups reflects improved kidney health, as several studies have documented a positive association between sulfhydryl groups and kidney health [56,77]. Lower senescence in the kidney exposed to MCFAs is associated with lower oxidative stress in these groups, as oxidative stress has been recognized as a direct inducer of cellular senescence [74,78].

Limitation of the study: the identification of only three targeted MCFAs in SO and OSO represents a limitation of the present study. This issue will be addressed in future investigations through a comprehensive fatty acid analysis incorporating short-, medium- and long-chain fatty acids in SO before and after the ozonation. Despite several similarities with humans, zebrafish exhibit many metabolic differences. For instance, unlike homeothermic (warm-blooded) humans, zebrafish are poikilothermic (cold-blooded) [22]; consequently, they exhibit differences in metabolic rate in response to environmental conditions [79]. In addition, zebrafish have a higher proportion of apolipoproteins (around 36% vs. ~10% in humans) and HDL-C/TC (%) (around 70% vs. ~25% in humans), and display distinct lipoprotein composition, including elevated triglycerides and reduced cholesterol esters in LDL [80]. Strikingly distinct from humans, zebrafish lack brown adipose tissue [79], which plays an important role in regulating obesity and related metabolic disorders [81]. Hence, the findings derived from zebrafish must be verified prudently in the context of their response to humans.

## 5. Conclusions

The LC/MS analysis confers the presence of three MCFAs (decanoic, octanoic, and hexanoic acids) in OSO. All detected MCFAs demonstrated substantial antioxidant activity to enhance HDL functionality, protect LDL, and increase the survivability of zebrafish embryos by protecting them from oxidative damage. The MCFAs curb CML-induced inflammation and oxidative stress, thereby rescuing zebrafish from acute death and swimming abnormalities. The MCFAs substantially counteracted dyslipidemia and hyperglycemia and augmented plasma antioxidant status, thereby protecting the liver and kidney from the stress imposed by CML. The study establishes the presence of MCFAs in OSO, suggesting them as one of the functional constituents responsible for diverse pharmacological properties. The present findings suggest the therapeutic potential of MCFAs in mitigating oxidative stress, inflammation and liver and kidney damage in zebrafish, suggesting probable use in human conditions. Nevertheless, additional preclinical and clinical studies are necessary to verify safety and efficacy in humans.

## Figures and Tables

**Table 1:** Concentration of decanoic acid, octanoic acid, and hexanoic acid in sunflower and ozonated sunflower oil quantified by HPLC analysis, and their physicochemical properties.

Compound	Molecular Formula (MW)	Concentration (mg/g of Oil)		Dissociation Constant [37] (pKa at 25 °C)	Melting Point [38] (°C)	Viscosity [39] (mPa·s)	Odor Threshold Low (ppm)
		SO (Sunflower Oil)	OSO (Ozonated Sunflower Oil)				
Decanoic acid	C_10_H_20_O_2_ (172.26)	1.23	2.88	4.90	31.5	4.30 at 50 °C	0.007
Octanoic acid	C_8_H_16_O_2_ (144.21)	ND	1.73	4.89	16.5	5.74 at 20 °C	0.008
Hexanoic acid	C_6_H_12_O_2_ (116.16)	ND	3.65	4.88	−3.4	3.23 at 20 °C	0.001

ND: Not detected/below the detection limit of the instrument.

## Data Availability

The data used to support the findings of this study are available from the corresponding author upon reasonable request.

## Supplementary Materials

The following supporting information can be downloaded at: https://www.mdpi.com/article/10.3390/antiox15050606/s1. Supplementary Table S1: Certificate of ozonated sunflower oil (OSO) quality analysis. Supplementary Table S2: Linear regression equations derived from reference hexanoic acid, octanoic acid and decanoic acid. Supplementary Table S3: Certificate of water quality analysis. Supplementary Material Section S1: HPLC methodology for the NPH derivatized samples. Supplementary Material Section S2: Methodology for the quantification of plasma lipid profile and hepatic function biomarkers. Supplementary Material Section S3: methodology for the detection of plasma oxidative and antioxidant variables. Supplementary Figure S1: HPLC chromatograms of NPH-derivatized standard MCFAs, SO and OSO.
